# LncRNA *H19*-Derived *miR-675-5p* Accelerates the Invasion of Extravillous Trophoblast Cells by Inhibiting *GATA2* and Subsequently Activating Matrix Metalloproteinases

**DOI:** 10.3390/ijms22031237

**Published:** 2021-01-27

**Authors:** Manabu Ogoyama, Akihide Ohkuchi, Hironori Takahashi, Dongwei Zhao, Shigeki Matsubara, Toshihiro Takizawa

**Affiliations:** 1 Department of Obstetrics and Gynecology, Jichi Medical University, 3311-1 Yakushiji, Shimotsuke-shi, Tochigi 329-0498, Japan; 99019mo@jichi.ac.jp (M.O.); okuchi@jichi.ac.jp (A.O.); hironori@jichi.ac.jp (H.T.); matsushi@jichi.ac.jp (S.M.); 2 Department of Molecular Medicine and Anatomy, Nippon Medical School, 1-1-5 Sendagi, Tokyo 113-8602, Japan; d-zhao@nms.ac.jp

**Keywords:** extravillous trophoblast, cell invasion, long non-coding RNA *H19*, *miR-675-5p*, GATA2, matrix metalloproteinase

## Abstract

The invasion of extravillous trophoblast (EVT) cells into the maternal decidua, which plays a crucial role in the establishment of a successful pregnancy, is highly orchestrated by a complex array of regulatory mechanisms. Non-coding RNAs (ncRNAs) that fine-tune gene expression at epigenetic, transcriptional, and post-transcriptional levels are involved in the regulatory mechanisms of EVT cell invasion. However, little is known about the characteristic features of EVT-associated ncRNAs. To elucidate the gene expression profiles of both coding and non-coding transcripts (i.e., mRNAs, long non-coding RNAs (lncRNAs), and microRNAs (miRNAs)) expressed in EVT cells, we performed RNA sequencing analysis of EVT cells isolated from first-trimester placentae. RNA sequencing analysis demonstrated that the lncRNA *H19* and its derived miRNA *miR-675-5p* were enriched in EVT cells. Although *miR-675-5p* acts as a placental/trophoblast growth suppressor, there is little information on the involvement of *miR-675-5p* in trophoblast cell invasion. Next, we evaluated a possible role of *miR-675-5p* in EVT cell invasion using the EVT cell lines HTR-8/SVneo and HChEpC1b; overexpression of *miR-675-5p* significantly promoted the invasion of both EVT cell lines. The transcription factor gene *GATA2* was shown to be a target of *miR-675-5p*; moreover, small interfering RNA-mediated *GATA2* knockdown significantly promoted cell invasion. Furthermore, we identified MMP13 and MMP14 as downstream effectors of *miR-675-5p*/*GATA2*-dependent EVT cell invasion. These findings suggest that *miR-675-5p*-mediated *GATA2* inhibition accelerates EVT cell invasion by upregulating matrix metalloproteinases.

## 1. Introduction

During early placentation, the invasion of extravillous trophoblast (EVT) cells into the maternal decidua and subsequent remodeling of the spiral arteries play crucial roles in the establishment of a successful pregnancy [1]. Insufficient EVT cell invasion often results in poor placentation leading to pregnancy complications such as preeclampsia and fetal growth restriction [1,2]. EVT cell invasion is highly orchestrated by a complex array of regulatory mechanisms, including angiogenic factors (e.g., VEGF, PGF, MMP2, and MMP9) [3,4], cytokines (e.g., CXCL10, CXCL12, IL4, IL6, and IL8) [3,5], immune cells (Th1/2/17 helper T cells, natural killer cells, and macrophages) [6,7], and cell adhesion molecules (e.g., CDH1 and CD44) [4,8,9]. These complex mechanisms are controlled by various gene regulatory and signaling networks.

Non-coding RNAs (ncRNAs) that are not translated into protein have recently attracted much attention for their ability to fine-tune gene expression at epigenetic, transcriptional, and post-transcriptional levels [10]. These ncRNAs can be classified into two main types: small ncRNAs (sncRNAs) with lengths of 20–50 nucleotides and long ncRNAs (lncRNAs) of >200 nucleotides [11]. Of the two types, sncRNAs, especially microRNAs (miRNAs), are also involved in the molecular mechanisms of EVT cell invasion [12]. However, little is known about the characteristic features of EVT-associated ncRNAs. In this study, we first performed RNA sequencing analysis of EVT cells isolated from the first-trimester placenta to elucidate gene expression profiles of both coding and non-coding transcripts (i.e., mRNAs, lncRNAs, and miRNAs) expressed in EVT cells. We found that the lncRNA *H19* and its derived miRNA *miR-675-5p* were enriched in EVT cells. The sncRNA *miR-675-5p* is generated from the lncRNA *H19*, which serves as a primary miRNA precursor of *MIR675* [13]. Previous studies have reported that *miR-675-5p* acts as a placental growth suppressor [14,15]. Additionally, some studies have suggested that *miR-675-5p* inhibits trophoblast cell proliferation [16,17]. However, no studies have investigated the involvement of *miR-675-5p* in the regulatory mechanisms of trophoblast cell invasion. This miRNA may also play a role in cancer cell invasion and metastasis [18,19]. Hence, we hypothesized that *miR-675-5p* plays a role in EVT cell invasion in the human placenta during early pregnancy. To test our hypothesis, we evaluated the role of *miR-675-5p* in EVT cell invasion using EVT cell lines. We found that *miR-675-5p* accelerated EVT cell invasion by direct suppressing the transcription factor gene *GATA2*. Furthermore, we revealed that matrix metalloproteinases (i.e., MMP13 and MMP14) were downstream effectors of *miR-675-5p*/*GATA2*-dependent EVT cell invasion.

## 2. Results

### 2.1. Identification and Characteristics of Genes Expressed in EVT Cells from RNA Sequencing

From the cDNA libraries of EVT and chorionic villous trophoblast (CVT) cells, 17,194 protein-coding genes (mRNAs), 4370 lncRNAs, and 1560 mature miRNAs were defined as expressed. A statistical summary of clean reads from RNA sequencing of sample libraries is provided in Table S5A. First, to assess whether isolated cells from the first-trimester placenta have the characteristics of EVT cells, we paid attention to 12 mRNAs previously reported to be trophoblast cell markers (Table S5C). Cytotrophoblast cells express *EGFR*, *TEAD4,* and *TP63*; EVT cells have lost these markers and instead express *HLA-G*, *MCAM*, *MYC*, and *CDH5* [20,21,22]. EVT cells are also characterized by the loss of genes encoding integrin subunits (*ITGA6* and *ITGB4*), and the gain of *ITGA1*, *ITGA5*, and *ITGB1* [23,24,25]. Six EVT cell marker genes, except for *CDH5*, were significantly upregulated in the isolated cells compared with CVT cells; five cytotrophoblast cell marker genes were significantly downregulated in the isolated cells (Table S5C). Our RNA-sequencing data of the expression of these trophoblast cell markers, except for *CDH5*, are consistent with previous reports on these expression changes at the mRNA level, suggesting that the isolated cells represent EVT cells.

**Figure 1:**
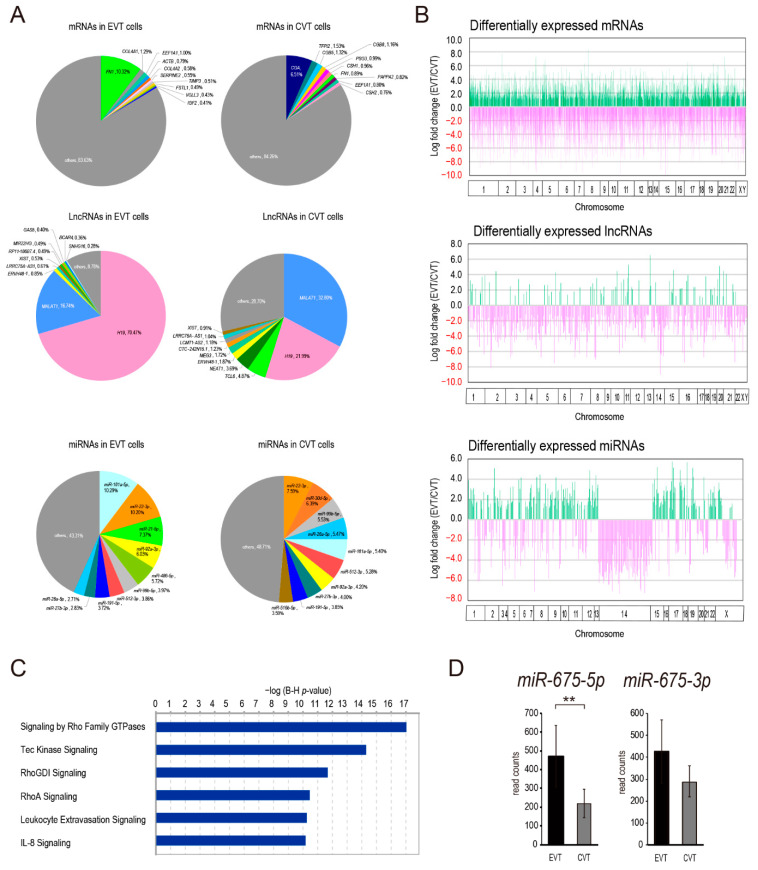
RNA-sequencing analysis of extravillous trophoblast (EVT) cells isolated from first-trimester human placentae. (**A**) Pie charts showing the top 10 most abundant genes (mRNAs, lncRNAs, and miRNAs) in EVT and chorionic villous trophoblast (CVT) cells, expressed as percentages. (**B**) The chromosomal distribution and expression of DEGs (mRNAs, lncRNAs, and miRNAs) across human chromosomes in EVT cells. The *y*-axis represents log2 fold change in gene expression. The *x*-axis represents chromosome number (1–22, X, and Y). The cell size of each chromosome is shown by the total number of DEGs contained in each chromosome. In each chromosome, DEGs are ordered along the *x*-axis according to their genomic position (left to right). (**C**) IPA canonical pathway analysis of EVT-associated DEGs. The most relevant canonical signaling pathways for DEGs are shown; the *x*-axis represents the −log of the *p*-value calculated using the Benjamini–Hochberg method. (**D**) RNA-sequencing analysis of *miR-675-5p* and *miR-675-3p* in EVT and CVT cells. The *y*-axis represents the mean of normalized counts. Data are presented as the mean ± standard deviation (SD) of the analysis of three independent samples; ** *q* < 0.01.

Differentially expressed genes (DEGs) were identified based on a *q* < 0.05 and an absolute log2 fold change (log FC) ≥ 1. Of 23,124 genes, 5092 mRNAs, 422 lncRNAs, and 401 mature miRNAs were identified as differentially expressed. Details of the DEGs expressed in EVT cells can be found in Tables S5–S8. The chromosomal distribution of the DEGs is also shown in Figure 1B. The most highly expressed mRNAs, lncRNAs, and miRNAs identified in EVT and CVT cells are shown in Figure 1A and Tables S6–S8. The most remarkable point of this RNA-sequencing study is that all DEGs (i.e., ten mRNAs, seven lncRNAs, and 83 miRNAs) located in chromosome 14q32.2, which contain the eutherian-specific imprinted *DLK1*-*DIO3* region [26], were significantly downregulated in EVT cells (Figure 1B and Table S9). In terms of imprinted genes (http://www.geneimprint.com/site/genes-by-species), 68 genes were found to be DEGs; the percentages of the maternally and paternally imprinted genes that were significantly downregulated in EVT cells were 63% (19 out of 30 maternal genes) and 89% (23 out of 29 maternal genes), respectively (Table S10).

Using Ingenuity Pathway Analysis (IPA), we performed Core Analysis to interpret our dataset of DEGs filtered by the IPA microRNA Target Filter tool (i.e., the dataset containing 351 miRNAs targeting 4783 mRNAs and lncRNAs). The top canonical pathways, as shown in Figure 1C were related to the reorganization of the actin cytoskeleton and modulation of cell adhesion/migration (e.g., signaling by Rho family GTPases and Tec kinase signaling). Most of the top enriched terms corresponding to Diseases/Functions based on significant activation z-score (absolute z-score > 2.0) were associated with invasion- or cancer-related functions (Table S11). The bioinformatics analysis revealed that DEG-containing molecular pathways/gene sets reflect the invasive differentiation process of trophoblast cells.

Another remarkable point of this RNA-sequencing study was that lncRNA *H19* was encoded by a DEG (*q* = 1.29 × 10^−9^, log FC = 2.98) and the most highly expressed lncRNA (70.47%) in EVT cells (Figure 1A and Table S7), whereas most of the differentially expressed lncRNAs (81%) were downregulated in EVT cells (Figure 1B). The high expression level of lncRNA *H19* in EVT cells revealed by RNA-sequencing was in good agreement with previous findings from single-cell RNA-sequencing and microarray experiments [27,28]. The lncRNA *H19*-derived miRNA *miR-675-5p* was relatively highly expressed in EVT cells, ranking 77th among the 401 differentially expressed miRNAs between EVT and CVT cells (Figure 1D). There was no significant difference in the expression of the other lncRNA *H19*-derived mature miRNA *miR-675-3p* between EVT and CVT cells (Figure 1D). We next focused on the lncRNA *H19*-derived *miR-675-5p* to investigate its role in EVT cell invasion.

### 2.2. miR-675-5p Accelerates EVT Invasion

**Figure 2:**
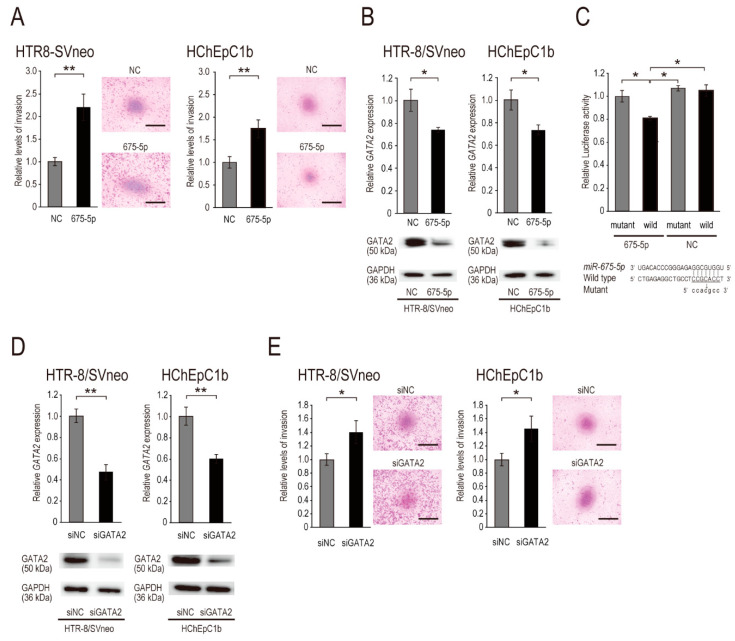
*miR-675-5p* accelerated the invasive ability of EVT cell lines via *GATA2* inhibition. (**A**) Transwell invasion assay of EVT cell lines (HTR-8/SVneo and HChEpC1b) transfected with a miR-675-5p (675-5p) or negative control (NC) mimic. The number of invasive cells is expressed as a percentage of the control value, and the invasion level of NC mimic was set at 1.0. (**B**,**C**) Validation of *GATA2* as a *miR-675-5p* target. (**B**) GATA2 mRNA (upper panel) and protein (lower panel) expression in EVT cell lines transfected with 50 nM 675-5p or NC mimic and cultured for 48 h. (**C**) *GATA2* 3′-UTR luciferase reporter assay. A reporter vector (pMIR-GATA2/wild or pMIR-GATA2/mut) and miR mimic (20 nM 675-5p or NC) were co-transfected into cells. The *Renilla* luciferase vector pRL-TK was used as the internal control. Luciferase expression levels in cells co-transfected with pMIR-GATA2/mut and 675-5p were defined as 1.0. Sequences of the putative target site of *miR-675-5p* in the 3′-UTR of *GATA2* and the mutation introduced into the *miR-675-5p* recognition site of the 3′-UTR in the reporter plasmid (lower panel). (**D**) Knockdown efficiency in EVT cell lines (HTR-8/SVneo and HChEpC1b) transfected with siGATA2. GATA2 mRNA and protein expression in EVT cell lines transfected with 20 nM siGATA2 or siNC and cultured for 24 h. (**E**) Transwell invasion assay with EVT cell lines transfected with siGATA2 or siNC. For the invasion assay, the number of invasive cells was expressed as a percentage of the control value, and the invasion level of siNC was set at 1.0. Normalization in qPCR was performed using 18S; GAPDH was used as the internal control in Western blotting. Data are presented as the mean ± SD of the results of three independent experiments. Student’s *t*-test (**A**,**B**,**D**,**E**) or Tukey’s test (**C**) was used to assess between-group differences; * *p* < 0.05, ** *p* < 0.01. Bars = 1 mm.

To assess the possible contribution of *miR-675-5p* to EVT cell invasion, we performed a Matrigel-coated Transwell invasion assay using the EVT cell lines HTR-8/SVneo and HChEpC1b. Following the transfection of *miR-675-5p* (675-5p) mimic into the EVT cell lines, *miR-675-5p* significantly promoted the invasion of EVT cells compared with the control (Figure 2A). In addition, we assessed the effect of *miR-675-5p* transfection on cell growth. However, no significant difference in cell growth was identified between 675-5p- and negative control (NC) mimic-transfected cells (Figure S1A).

### 2.3. miR-675-5p Targets GATA2 Directly

Next, we sought to identify the target genes of *miR-675-5p* using a target prediction program, TargetScanHuman 7.2; 1352 target gene candidates were obtained. Among these overrepresented genes, we focused on 12 genes previously reported to be tumor/invasion suppressor genes (i.e., *BHLHE41* (also known as *SHARP1*), *CDKN2A*, *CHMP1A*, *DDB2*, *DRD2*, *EMILIN1*, *GATA2*, *ING5*, *LCN2*, *NOG*, *RUNX1*, and *SMAD5*) [19,29,30,31,32,33,34,35,36,37,38,39] (Table S4) and performed quantitative polymerase chain reaction (qPCR) to investigate whether these suppressor genes are downregulated by the overexpression of *miR-675-5p* in HTR-8/SVneo cells. Among the 12 genes, *BHLHE41*, *CHMP1A*, *GATA2*, and *NOG* were significantly downregulated in *miR-675-5p*-overexpressing cells compared with control cells (Figure 2B and Figure S1B). Furthermore, we used a luciferase reporter assay to determine whether these downregulated genes were direct targets of *miR-675-5p*. The overexpression of *miR-675-5p* significantly decreased luciferase activity in HTR-8/SVneo cells co-transfected with pMIR-GATA2/wild compared with the NC, but not in cells co-transfected with pMIR-GATA2/mut (Figure 2C), a reporter plasmid in which the putative *miR-675-5p* recognition site in the *GATA2* 3′-untranslated region (3′-UTR) was mutated (Figure 2C). Luciferase activities in cells co-transfected with pMIR-REPORT vectors into which the 3′-UTRs of the candidate genes *BHLHE41*, *CHMP1A*, and *NOG* were inserted were not significantly suppressed compared with the control cells, suggesting that they may be regulated by indirect target genes of *miR-675-5p* (Figure S1C). We also examined the protein levels of GATA2 in EVT cell lines overexpressing *miR-675-5p*. As shown in Figure 2B, *miR-675-5p* markedly downregulated the protein levels of GATA2 in these cell lines. Taken together, these results demonstrate that the transcription factor gene *GATA2* is a target of *miR-675-5p*.

### 2.4. miR-675-5p-Mediated GATA2 Inhibition Accelerates EVT Cell Invasion by Upregulating MMP13 and MMP14

To determine whether *miR-675-5p*-mediated *GATA2* inhibition accelerates EVT cell invasion, we analyzed invasion by cells in which *GATA2* was downregulated via small interfering RNA (siRNA)-mediated knockdown (designated as siGATA2). First, we evaluated the efficiency of siGATA2 using qPCR and Western blotting. In EVT cell lines, siGATA2 inhibited the expression of GATA2 mRNA and protein (Figure 2D). Next, we assayed the invasion of EVT cells treated with siGATA2. Following *GATA2* knockdown, siGATA2 significantly promoted the invasion of these cells compared with the control (Figure 2E), suggesting that GATA2 negatively regulates EVT cell invasion.

**Figure 3:**
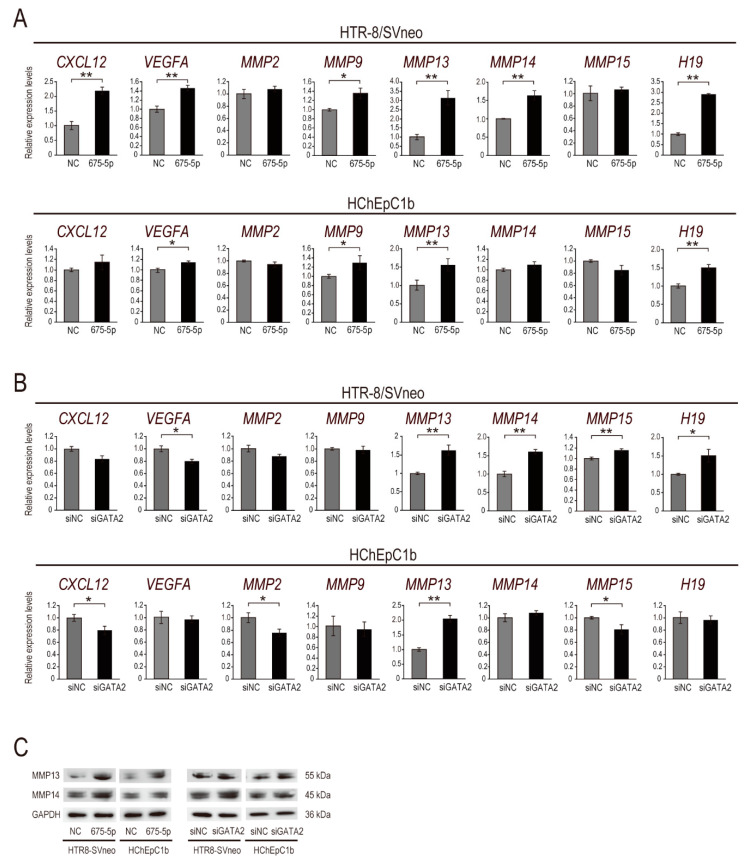
*miR-675-5p*-mediated *GATA2* inhibition upregulated MMP13 and MMP14. (**A**) qPCR analysis of genes associated with cell invasion in EVT cell lines (HTR-8/SVneo and HChEpC1b) transfected with 50 nM *miR-675-5p* (675-5p) or negative control (NC) mimic and cultured for 48 h. (**B**) qPCR analysis of genes associated with cell invasion in EVT cell lines transfected with 20 nM siGATA2 or siNC and cultured for 24 h. (**C**) Western blot of MMP13 and MMP14. MMP13 and MMP14 expression in EVT cell lines transfected with 675-5p or a NC mimic (left panel); MMP13 and MMP14 expression in cells transfected with siGATA2 or siNC (right panel). Normalization in qPCR was performed using 18S; GAPDH was used as the internal control in Western blotting. Data are presented as the mean ± SD of the results of three independent experiments. * *p* < 0.05, ** *p* < 0.01; Student’s *t*-test.

Next, we sought to investigate *miR-675-5p*/*GATA2* axis-regulated downstream effectors in EVT cell invasion and focused on seven genes (i.e., *CXCL12*, *VEGFA*, *MMP2*, *MMP9*, *MMP13*, *MMP14*, and *MMP15*) previously reported to be associated with cell invasion [3,5,40,41,42]. To evaluate whether these candidate genes are upregulated in both *miR-675-5p*-overexpressing and siGATA2-transfected EVT (HTR-8/SVneo and HChEpC1b) cells compared with control cells, we investigated their expression levels using qPCR (Figure 3A,B). Among the seven genes selected, *miR-675-5p* significantly increased the expression levels of *CXCL12*, *VEGFA*, *MMP9*, *MMP13*, and *MMP14* in both cell lines, but not those of *CXCL12* and *MMP14* in HChEpC1b cells (Figure 3A). Furthermore, siGATA2 significantly increased the expression levels of *MMP13* and *MMP14* in HTR8/SVneo cells, as well as the expression of *MMP13* in HChEpC1b cells (Figure 3B). Based on the qPCR results, we investigated the protein levels of MMP13 and MMP14 in *miR-675-5p*-overexpressing and siGATA2-transfected EVT cells. Both *miR-675-5p* mimic and siGATA2 treatments markedly induced protein expression of MMP13 and MMP14 in HTR8/SVneo cells (Figure 3C). Thus, we focused on MMP13 and MMP14 as *miR-675-5p*/*GATA2* axis-regulated downstream effectors in EVT cell invasion. In addtion, both *miR-675-5p* mimic and siGATA2 treatments promoted the expression of the lncRNA *H19* in EVT cells, suggesting that the lncRNA *H19/miR-675-5p*/*GATA2* axis may constitute a positive feedback loop (Figure 3A,B).

**Figure 4:**
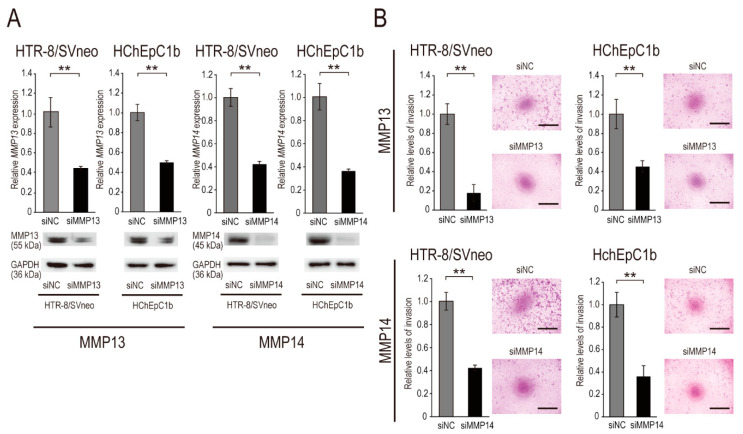
MMP14 and MMP13 inhibition attenuated the invasive ability of EVT cell lines. (**A**) Knockdown efficiency in EVT cell lines (HTR-8/SVneo and HChEpC1b) transfected with siMMP13 and siMMP14. MMP13 mRNA and protein (left panel), and MMP14 mRNA and protein (right panel) expression in EVT cell lines transfected with 20 nM siMMP13, siMMP14, or siNC and cultured for 24 h. Normalization in qPCR was performed using 18S; GAPDH was used as the internal control in Western blotting. (**B**) Transwell invasion assay of EVT cell lines transfected with siMMP13 and siMMP14. For the invasion assay, the number of invasive cells was expressed as a percentage of the control value, and the invasion level of siNC was set at 1.0. Data are presented as the mean ± SD of the results of three independent experiments. ** *p* < 0.01; Student’s *t*-test. Bars = 1 mm.

To evaluate whether MMP13 and MMP14 accelerate EVT cell invasion, we analyzed invasion by cells in which *MMP14* and *MMP13* were downregulated by siRNA mediated knockdown (designated siMMP13 and siMMP14, respectively). First, we assessed the efficiency of siMMP13 and siMMP14 using qPCR and Western blotting in EVT cells and found that siMMP13 and siMMP14 significantly inhibited both the mRNA and protein levels of MMP13 and MMP14, respectively (Figure 4A). Next, we assayed the invasion of EVT cells treated with siMMP13 and siMMP14. Following *MMP13* knockdown, siMMP13 significantly attenuated the invasive ability of HTR8/SVneo and HChEpC1b cells (Figure 4B). Additionally, siMMP14 significantly inhibited the invasive ability of these EVT cells compared with the control values (Figure 4B). Taken together, these findings suggest that both MMP13 and MMP14 promote EVT cell invasion as downstream effectors of the *miR-675-5p*/*GATA2* axis.

## 3. Discussion

We performed RNA-sequencing analysis of EVT cells isolated from first-trimester human placentae to profile the gene expression signatures of mRNAs as well as ncRNAs (i.e., miRNAs and lncRNAs). We identified approximately 6000 DEGs between EVT and CVT cells. Bioinformatics analysis revealed that DEG-containing signaling pathways/gene sets are associated with the migratory/invasive phenotype of trophoblast cells. This EVT phenotype revealed by RNA-sequencing is consistent with previous findings from microarray experiments [27,43]. Single-cell RNA-sequencing technology has recently revealed unique gene signatures of EVT cells in healthy pregnancy and preeclampsia [28,44,45]. Although the regulatory mechanisms of ncRNAs in the EVT-associated lncRNA-miRNA-mRNA network will require further elucidation, the present study will be a valuable resource for future ncRNA research on EVT cells.

We originally hypothesized that *miR-675-5p* may play a role in EVT cell invasion during early pregnancy. In the present study, we showed that *miR-675-5p*, an EVT-associated miRNA, was involved in the regulation of EVT cell invasion. Our data further revealed that *miR-675-5p* accelerated EVT cell invasion by direct suppressing *GATA2* and subsequently activating MMP13 and MMP14.

Regarding the function of lncRNA *H19*-derived *miR-675-5p* in trophoblast cells, although some researchers have reported on its role in cell proliferation, we could not find a significant association between *miR-675-5p* and the proliferation of EVT cell lines in this study (Figure S1A). Gao et al. showed that *miR-675-5p* inhibited trophoblast cell proliferation via direct inhibition of *NOMO1* using JEG3 cells [16], whereas Keniry et al. demonstrated that *miR-675-5p* attenuated trophoblast cell proliferation by suppressing *Igfr1* using mouse trophoblast stem cell models [17]. By contrast, there are few reports on the role of *miR-675-5p* in trophoblast cell invasion. This work is the first to demonstrate that the enrichment of *miR-675-5p* in EVT cells promoted EVT cell invasion. The function of *miR-675-3p*, the other strand from *MIR675*, in trophoblast cells remains unknown.

Within the context of trophoblast cell invasion, many studies have reported that various miRNAs and their target genes are associated with trophoblast cell invasion [12,46,47]. In this study, *miR-675-5p* promoted trophoblast cell invasion by direct suppressing *GATA2*. The transcription factor GATA2 belongs to the mammalian GATA family of zinc-finger transcription factors, which consists of six proteins (GATA1–6) involved in a variety of physiological and pathological processes [48]. GATA2 and GATA3 are trophoblast intrinsic master regulators of gene expression in all trophoblast progenitors [49]. Both GATA2 and GATA3 are constitutively expressed in all trophoblast cell types in the developing human placenta; they coordinate gene regulatory networks to generate specialized trophoblast cell types during placentation. Furthermore, dual *Gata2*/*Gata3* conditional gene deletion study revealed the importance of functional redundancy of Gata2 and Gata3 in mouse trophoblast development [50]. Since the present study focused on the regulatory role of *miR-675-5p*/*GATA2* axis in EVT cell invasion, the possible involvement of *miR-675-5p*/*GATA2* axis in the GATA2/GATA3-dependent transcriptional mechanisms in EVT cells requires further investigation. Although various studies have investigated the role of *miR-675-5p* in cancer cell invasion [18,51,52,53], the promotion and inhibition of cancer cell invasion by *miR-675-5p* remain controversial. Zheng et al. reported that *miR-675-5p* promoted glioma cell invasion, migration, and proliferation via negative regulation of the tumor suppressor gene *RB1* [51]. Zhou et al. showed that *miR-675-5p* accelerated cell invasion, migration, and proliferation of esophageal squamous cell carcinoma cells by inhibiting *REPS2*, which is involved in growth factor signaling [18]. In that sense, *RB1* and *REPS2* may be potential *miR-675-5p* target genes in EVT cell invasion. In contrast, He et al. reported that *miR-675-5p* suppressed cell invasion, migration, and proliferation of non-small cell lung cancer cells by targeting the proto-oncogene gene *GPR55* [52]. Additionally, *miR-675-5p* has been shown to inhibit cell invasion, migration, and the proliferation of papillary thyroid cancer cells by suppressing *MAPK1* [53]. It is likely that the dual roles of *miR-675-5p* in cancer cell invasion depend on the type of cancer, as target mRNA and protein levels are differentially modulated.

Trophoblast cell invasion is tightly controlled by triggering or inhibiting various signaling pathways, including MAPK, PI3K/AKT, JAK-STAT, Wnt, FAK and Rho/ROCK, and TGFB superfamily signaling [54,55]. Twenty-six MMPs are expressed in human EVT cells [56,57]. MMPs, especially MMP2 and MMP9, are key enzymes participating in trophoblast cell invasion via these pathways [3,54,58]; MMP2 and MMP9 are abundantly expressed in EVT cells [59,60]. In this study, *MMP2* expression was not regulated by *miR-675-5p* in EVT cell lines (Figure 3A). Although *MMP9* was significantly upregulated by *miR-675-5p* in EVT cells, *MMP9* expression was not altered by *GATA2* knockdown (Figure 3A,B). Thus, *MMP9* is regulated by other target genes of *miR-675-5p*. Although Luan et al. demonstrated that CCR7-mediated GATA2 suppression attenuated the invasive ability of trophoblast cell lines (JAR and JEG3) via MMP2 [61], there are few reports of other MMPs regulated by GATA2 in EVT cell invasion. The present study identified MMP13 and MMP14 as novel downstream effectors of GATA2 in trophoblast cells. MMP13 (also known as collagenase-3) is secreted into the extracellular space as an inactive proenzyme (pro-MMP13); its activation requires cleavage of pro-MMP13 by MMP3 and MMP14 [62,63]. Active MMP13 initiates activation of pro-MMP9 [64] and degrades native fibrillar collagen (e.g., collagen type 1/2) [40,65]. Although MMP13 enhances the invasive capacity of cancer cells [66,67,68], the function of MMP13 in trophoblast cells remains largely undetermined. MMP14 (also known as MT1-MMP), a member of the membrane-type MMP subfamily, activates pro-MMP2, pro-MMP8, and pro-MMP13 [62,69,70], and cleaves several cell adhesion molecules (e.g., collagen type-1/2/3 and CD44), which accelerates cell invasion [71,72,73]. MMP14 is highly expressed in the human placenta, especially in EVT cells in the first-trimester [74,75,76]. Wang et al. showed that MMP14 accelerated EVT cell invasion via Notch1 and PI3K/AKT signaling using HTR-8/SVneo cells [77]. Majali-Martinez et al. showed that MMP14 promoted the invasion of primary trophoblast cells [78]. Our findings of EVT cell invasion following MMP14 activation are consistent with these previous studies. Given that MMP14 activates pro-MMP13 on the cell surface in the presence of active MMP2 [62], EVT cell invasion may be synergistically enhanced by a combination of MMP13 and MMP14.

In terms of RNA sequencing, we found significant downregulation of DEGs in the chromosome region 14q32.2 (i.e., the *DLK1-DIO3* region). The *DLK1*-*DIO3* cluster contains three paternally expressed protein coding genes (*DLK1*, *RTL1*, and *DIO3*) and multiple maternally expressed ncRNAs (e.g., *MEG3* and 54 clustered miRNA precursors (designated as C14MC miRNAs)) [26]. These imprinted genes are inversely regulated by differentially methylated regions [79,80,81]. Liu et al. reported that the degree of activation of the *Dlk1*-*Dio3* region is correlated with pluripotency levels of mouse stem cells [82]. Several C14MC miRNAs may form a feedback loop by suppressing PRC2 formation for gene methylation of this region, resulting in the activation of all genes encoded by this region in pluripotent stem cells. In this study, the inactivation of the *DLK1*-*DIO3* region in EVT cells suggests the loss of stemness of CVT cells and subsequent differentiation into EVT cells. Greife et al. reported epigenetic silencing across the *DLK1*-*DIO3* imprinted gene cluster due to aberrant epigenetic regulation (i.e., unique changes in DNA methylation and repressive histone modifications), which resulted in the simultaneous downregulation of oppositely expressed and imprinted genes of the region in urothelial carcinoma [83]. The epigenetic regulatory mechanism and functional role(s) of the gene downregulation across the *DLK1*-*DIO3* region in EVT cells remain to be elucidated. Although C14MC miRNAs are expressed predominantly in human placental trophoblast cells (i.e., placenta-associated miRNAs), there are two other placenta-associated miRNA clusters in chromosome 19: the chromosome-19 miRNA cluster (designated as C19MC; 46 miRNA precursors) and the miR-371-3 cluster (three miRNA precursors in the vicinity of C19MC) [84,85]. The C19MC miRNAs are paternally imprinting genes [86]. As described above, the expression levels of most C14MC miRNAs (83 out of 99 mature miRNAs) were significantly downregulated in EVT cells (Figure 1B and Table S9), whereas the expression levels of most of the C19MC and miR-371-3 cluster miRNAs did not differ significantly between EVT and CVT cells, although a decreasing tendency was observed for EVT cells (data not shown).

This study has some limitations. We employed two EVT cell lines (HTR8/SVneo and HChEpC1b) for in vitro functional analysis since the numbers of obtainable first-trimester placentas after legal abortions were being limited in our institutes. Therefore, future work should perform using primary first-trimester EVT cells. In addition, the specific roles of lncRNA *H19*, except for its role as a primary miRNA of *MIR675*, in EVT cells are still unknown. LncRNA *H19* is located in an imprinted region of human chromosome 11, along with the *IGF2* gene [87]. *H19* and *IGF2* are reciprocally imprinted (maternally and paternally expressed, respectively) and regulated by the *H19* differentially methylated region (H19-DMR). *H19* and *IGF2* are important for proper placental development function [87,88]. There are a few reports on lncRNA *H19* promoting EVT cell invasion by acting as an endogenous miRNA sponge [89,90]. The dysregulation of lncRNA *H19* and *miR-675-5p* in EVT cells during first-trimester pregnancy may cause inadequate EVT cell differentiation, invasion, and subsequent placenta dysfunction [90]. Epimutation at the H19-DMR in humans results in congenital imprinting disorders (i.e., Beckwith-Wiedemann and Silver–Russell syndromes) [91,92]. Placental epimutation at H19-DMR has also been reported among pregnancy complication cases [93]. Besides, during the isolation process of EVT cells for RNA-sequencing, each step may affect the expression signatures of EVT cells. Thus, it would be helpful to compare the results from RNA-sequencing with those from in vivo gene expression analysis (e.g., in situ hybridization) for the proper interpretation of the gene expression signatures.

**Figure 5:**
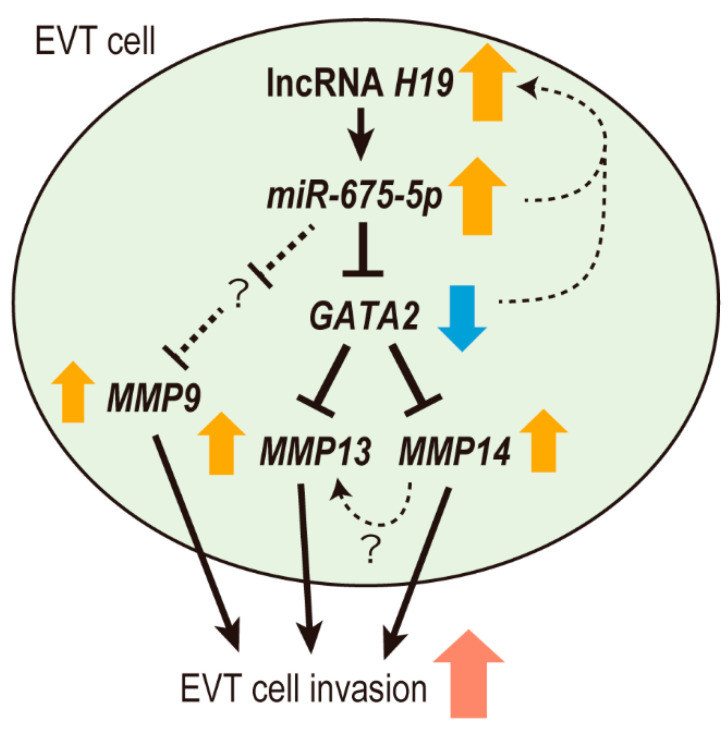
Proposed model for *miR-675-5p*/*GATA2*-mediated EVT cell invasion. *miR-675-5p*-mediated *GATA2* inhibition upregulates *MMP14* and *MMP13*, resulting in the promotion of EVT cell invasion. *miR-675-5p* also upregulates *MMP9* in EVT cells by other target genes of *miR-675-5p*. Moreover, *miR-675-5p*/*GATA2* promotes *H19* expression, suggesting the existence of a *H19*-*miR-675-5p*/*GATA2* positive feedback system.

In summary, we performed RNA-sequencing analysis of EVT cells isolated from the first-trimester placenta and found significant downregulation of DEGs in the chromosome region 14q32.2 (i.e., the *DLK1-DIO3* region). RNA-sequencing analysis also revealed that the lncRNA *H19* and its derived miRNA *miR-675-5p* were abundantly expressed in EVT cells. We evaluated the possible role of *miR-675-5p* in EVT cell invasion using EVT cell lines. In vitro analysis demonstrated that the lncRNA *H19*-derived *miR-675-5p* accelerated EVT cell invasion by direct suppressing *GATA2*. Moreover, MMP13 and MMP14 were shown to be downstream effectors of *miR-675-5p*/*GATA2*. Thus, our study suggests that the *miR-675-5p*-*GATA2*-MMP14/MMP13 axis may play an important role in EVT cell invasion (Figure 5).

## 4. Materials and Methods

### 4.1. Culture of Cell Lines

HTR-8/SVneo and HChEpC1b cells were used as human EVT model cell lines [94,95]. The HTR-8/SVneo and HChEpC1b cells were maintained in RPMI-1640 medium (Wako, Osaka, Japan) supplemented with 5% and 10% fetal bovine serum (FBS; Japan Bio Serum, Hiroshima, Japan), respectively (37 °C, 5% CO_2_). The immortalized human lymphocyte cell line Jurkat was employed for the construction of luciferase reporter vectors; Jurkat cells were cultured in RPMI1640 medium supplemented with 10% FBS (37 °C, 5% CO_2_) [96].

### 4.2. Trophoblast Cell Collection

Human placentae from pregnant women who provided informed consent were obtained using protocols approved by the Jichi Medical University Ethics Committee and the Nippon Medical School Ethics Committee. First-trimester placental tissues (seven weeks of gestation, *n* = 3) were obtained after legal abortions. The gestational age was determined by the last menstrual period and confirmed by ultrasound measurement in the first-trimester of pregnancy.

EVT cells growing from explanted human chorionic villi were isolated as described previously [8,97]. Briefly, placental tissues were washed with RPMI-1640 and dissected to remove decidual tissues and fetal membrane. Small fragments of chorionic villi were teased apart and soaked in culture medium (RPMI-1640 containing 10% FBS and 100 U/mL Penicillin-Streptomycin (Gibco, Carlsbad, CA, USA)). Villous fragments were then minced into fine pieces with surgical blades. The fine pieces of villi were placed in collagen type 1-coated dishes (cat. no. 4020-010; Iwaki, Tokyo, Japan) for 4 h. After 48 h, outgrowth cells from adherent villous tips were dispersed using TrypLE Express (cat. no. 12604-021; Gibco, Carlsbad, CA, USA), passed through a nylon strainer with pore diameter 40 μm (cat. no. 352340; Becton Dickson, Franklin Lakes, NJ, USA) to remove the chorionic villous parts, and placed again in collagen type 1-coated dishes. Following the removal of nonadherent cells and debris by washing with phosphate-buffered saline (PBS), residual cells were considered isolated EVT cells. The fine pieces of villous fragments were defined as first-trimester chorionic villous trophoblast (CVT) cells; they had cytotrophoblast cells, syncytiotrophoblast, and sparse stromal cells and fetal endothelial cells.

### 4.3. RNA Sequencing and Data Analysis

Total RNA was extracted from each sample using RNAiso Plus (cat. no. 9109; Takara Bio, Shiga, Japan). For small RNA sequencing, the TruSeq Small RNA Sample Prep Kit-Set A (cat. no. RS-200-0012; Illumina, San Diego, CA, USA) was used to prepare small RNA-sequencing libraries, according to the manufacturer’s instructions. Briefly, 1 µg of total RNA was processed for adaptor ligation and subsequent cDNA amplification. A 145–160-bp size selection was then performed on a 6% polyacrylamide gel. For mRNA/lncRNA sequencing, libraries were initially prepared with 1 μg of total RNA. Following the removal of ribosomal RNA, the remaining RNA was fragmented and used for cDNA library construction (i.e., cDNA synthesis, adenylation of the 3′ end, subsequent adapter ligation, and cDNA amplification) with the TruSeq Stranded Total RNA Library Prep Kit (cat. no. RS-122-2301; Illumina, San Diego, CA, USA) according to the manufacturer’s instructions. Indexed cDNA libraries were pooled at an equimolar ratio and used at a final concentration of 10–16 pM with 1% PhiX Control. The miRNA and mRNA/lncRNA sequencing analyses were carried out on an Illumina MiSeq system using MiSeq v2 Reagent Kit 50cycle (cat. no. MS-102-2001; Illumina, San Diego, CA, USA) and MiSeq v2 Reagent Kit 300cycle (cat. no. MS-102-2002; Illumina, San Diego, CA, USA), respectively.

The 26 bp single-end reads for miRNA sequencing and the 151 bp paired-end reads for mRNA/lncRNA sequencing were obtained as fastq files and imported into CLC Genomics Workbench (version 8.0.1; Qiagen, Venlo, The Netherlands) for the analysis of raw data, including trimming, filtering, and the cleaning up of contaminated reads based on default parameters (a minimum quality score of 0.05 (equivalent to Phred quality score of 30)). Clean 26 bp and 151 bp reads were aligned to the human genome sequence retrieved from the Ensembl database (Assembly GRCh38.p13, database version 82.38 and 101.38) and the microRNA database miRbase (release 21), respectively, using the CLC Genomics Workbench “Map Reads to Reference” tool (length fraction = 0.95 and similarity fraction = 0.9).

Normalization of gene expression data was performed according to the trimmed mean of M-values (TMM) method with the edgeR package (version 3.3.8; Bioconductor, https://www.bioconductor.org/). Statistically significant differentially expressed genes were identified using edgeR [98]. Genes with a false discovery rate (FDR)-corrected *p*-value (*q*-value) of < 0.05 and an absolute log2 fold change (log FC) ≥ 1 were considered significantly differentially expressed (i.e., DEGs) [99,100]. The FDR was adjusted using the Benjamini–Hochberg procedure. Sequencing data are available from the NCBI GEO database (GEO Accession No. GSE163651; https://www.ncbi.nlm.nih.gov/geo/).

### 4.4. Transfection of RNA Oligos

For miRNA transfection analysis, miRNA mimic oligos for *hsa-miR-675-5p* (designated as 675-5p mimic) and *cel-miR-239b-5p* as a negative control (NC; designated as NC mimic) were purchased from Ajinomoto Bio-Pharma (Osaka, Japan). For mRNA-knockdown analysis, the following siRNA oligos were employed: siRNA for *GATA2* (siGATA2), sense: 5′-UUCUUGGACUUGUUGGACAUCUUCC-3′ and antisense: 5′-GGAAGAUGUCCAACAAGUCCAAGAA-3′; siRNA for *MMP13* (siMMP13), sense: 5′-GGAGAUAUGAUGAUACUAATT-3′ and antisense: 5′-UUAGUAUCAUCAUAUCUCCTT-3′; siRNA for *MMP14* (siMMP14), sense: 5′- CCAGAAGCUGAAGGUAGAATT-3′ and antisense: 5′- UUCUACCUUCAGCUUCUGGTT-3′; and nonspecific control siRNA (siNC), sense: 5′-UUCUCCGAACGUGUCACGUTT-3′ and antisense: 5′-ACGUGACACGUUCGGAGAATT-3′ (Ajinomoto Bio-Pharma). Each miRNA and siRNA oligo (at final concentrations of 50 nM and 20 nM, respectively) was transfected into cells using Lipofectamine 2000 (cat. no. 11668019; Invitrogen, Waltham, MA, USA) at 37 °C for 4 h.

### 4.5. Quantitative Polymerase Chain Reaction (qPCR)

Total RNA was isolated from the samples using RNAiso Plus. Then, qPCR was performed with the Applied Biosystems 7300 Real-Time PCR System (Waltham, MA, USA) according to the manufacturer’s instructions. TB Green Premix Ex Taq (cat. no. RR420; Takara Bio, Shiga, Japan) was employed for the quantitative analysis of mRNA level. To normalize the expression levels of mRNAs, 18S ribosomal RNA (designated as 18S) was used. Three independent analyses of the expression levels of each mRNA and miRNA were performed. Primers for the lncRNA *H19* and mRNAs are shown in Table S1.

### 4.6. Western Blotting

Cell lysates were obtained using mammalian protein extraction reagent (cat. no. 78501; Thermo Fisher Scientific, Waltham, MA, USA) supplemented with 1% Halt protease inhibitor cocktail (cat. no. 78429; Thermo Fisher Scientific, Waltham, MA, USA). Twenty micrograms of cell lysate protein were separated using Mini-PROTEAN TGX gels (4–20% gel: cat. no. 456-1094; Bio-Rad, Hercules, CA, USA) with a PowerPac Basic power supply (200 V, 30 min) (Bio-Rad, Hercules, CA, USA). The proteins were then transferred onto polyvinylidene fluoride membranes using Trans-Blot Turbo Mini 0.2 µm PVDF Transfer Packs (cat. no. 1704156; Bio-Rad, Hercules, CA, USA) with the Trans-Blot Turbo Transfer System (Bio-Rad, Hercules, CA, USA). Blotted proteins were incubated with a primary antibody (rabbit anti-GATA2 (cat. no. 11103-1-A; Proteintech, Rosemont, IL, USA), rabbit anti-MMP13 (cat. no. 18165-1-AP; Proteintech, Rosemont, IL, USA), rabbit anti-MMP14 (cat. no. 14552-1-AP; Proteintech, Rosemont, IL, USA), or mouse anti-GAPDH (cat. no. 60004-1-IG; Proteintech, Rosemont, IL, USA)) at room temperature for 2 h, followed by incubation with horseradish peroxidase-conjugated anti-mouse (cat. no. SA00001-1; Proteintech, Rosemont, IL, USA) or anti-rabbit (cat. no. HAF017; R&D systems, Minneapolis, MN, USA) secondary antibody at room temperature for 1 h. Signals were detected with Clarity Western ECL Substrate (cat. no. 1705060; Bio-Rad, Hercules, CA, USA) and visualized using an Amersham Imager 680 (GE Healthcare, Chicago, IL, USA).

### 4.7. Cell Invasion Assay

The cell invasion assay was performed as described previously [8]. Briefly, growth factor-reduced BD Matrigel (cat. no. 354230; Becton Dickinson, Franklin Lakes, NJ, USA) was coated onto cell culture inserts (Falcon Transparent polyethylene terephthalate (PET) Membrane/24 well, 8.0 µm pore size; cat. no. 353097; Corning, NY, USA). The Matrigel concentration was 200 μg/mL (2%). These upper chambers were set onto the lower chambers (Falcon 24-well tissue-culture (TC)-treated Cell Polystyrene Permeable Support Companion Plate; cat. no. 353504; Corning, NY, USA). The cells were transfected with miRNA mimics or the siRNAs described above. After transfection for 24 h, cells (3.5 × 10^4^ HTR-8/SVneo or 7.0 × 10^4^ HChEpC1b) in 250 μL of each culture medium without FBS were placed in the upper chamber. Then, 800 µl of each culture medium with FBS (5% for HTR-8/SVneo; 10% for HChEpC1b) was added to the lower chamber. After incubation for 24 h (HTR-8/SVneo) or 48 h (HChEpC1b), the non-invading cells at the top of each Transwell chamber were scraped off using cotton swabs. The invading cells at the bottom of the membrane were fixed with 2.5% glutaraldehyde (Wako, Osaka, Japan)-PBS, stained with 0.5% crystal violet (Wako, Osaka, Japan), and counted manually using a light microscope (KX4, Olympus, Tokyo, Japan) under 200× (HTR-8/SVneo) or 100× (HChEpC1b) magnification. The average numbers of invading cells in five areas were counted to reflect invasive ability. Three invasion assays were conducted under each experimental condition.

### 4.8. Cell Proliferation Assay

Evaluation of cell proliferation ability was performed using a CellTiter-Glo Luminescent Cell Viability Assay kit (cat. no. G7571; Promega, Madison, WI, USA). Briefly, 7500 HTR-8/SVneo or 15,000 HChEpC1b cells were placed in 96-well plates for 24 h and transfected with 675-5p or NC mimic (50 nM). After transfection for 24, 48, and 72 h, the cells were lysed using a reagent from the CellTiter-Glo Luminescent Cell Viability Assay kit, and the ATP activities of the cell lysates were measured using the Glomax-Multi Detection System (Promega, Madison, WI, US). ATP activity was measured five times for each experimental condition.

### 4.9. In Silico Prediction of miRNA Target Genes

The TargetScanHuman database (release 7.2; http://www.targetscan.org/vert_72/; accessed on 7 May 2018) was employed to predict miRNA target genes.

### 4.10. Luciferase Reporter Assay

The luciferase reporter assay for *GATA2* was carried out as follows. To construct a reporter plasmid, we first cloned the 3′-UTR of human *GATA2* into the pMIR-REPORT vector (Applied Biosystems, Waltham, MA, USA). Total RNA isolated from Jurkat cells was reverse-transcribed to cDNA using PrimeScript reverse transcriptase (cat. no. 2680A; Takara Bio, Shiga, Japan). The portion of the *GATA2* 3′-UTR (327 bp), including the target candidate sequence of *miR-675-5p* was then amplified from the cDNA with the following primers: GAGGGAGCTCACCCTTAGCAGCCCAGCAT (*Sac*I site underlined) and ACGCACGCGTCACCAAGTCTCCAAGTCCTTGTT (*Mlu*I site underlined). After sequence verification, the *GATA2* 3′-UTR (327 bp) was cloned into pMIR-REPORT via the *Sac*I and *Mlu*I restriction sites. This final construct was designated as pMIR-GATA2/wild. To construct a reporter plasmid with a mutated *miR-675-5p* recognition site for the *GATA2* 3′-UTR (Figure 2C), an inverse PCR method was used [101]. The primers used for the inverse PCR were as follows: *GATA2* 3′-UTR-mutation, forward: tgagaggctgcctccacgcctgaccgctgcCCAGGT, and reverse: gcagcggtcaggcgtggaggcagcctctcaGCGGTG (the complementary sequence is shown in lowercase). PCR amplification was carried out using the previously cloned vector ZERO Blunt TOPO containing the *GATA2* 3′-UTR. Plasmid DNA was digested by *Dpn*I. Amplified DNA was transformed into *Escherichia coli* (One Shot TOP10: cat. no. C404003; Thermo Fisher Scientific, Waltham, MA, USA). After sequence verification, the mutated 3′-UTR sequence was cloned into pMIR-REPORT via the *Sac*I and *Mlu*I restriction sites. The final construct was designated as pMIR-GATA2/mut.

For the reporter assay, HTR-8/SVneo cells were transfected with pMIR-GATA2/wild or pMIR-GATA2/mut and the control vector pRL-TK (*Renilla* luciferase expression plasmid), together with 20 nM 675-5p or NC mimic using Lipofectamine 2000 in 24-well plates. At 24 h after transfection, luciferase assays were performed using the Dual Luciferase Reporter Assay System (Promega, Madison, WI, USA). Firefly luciferase (from the pMIR-REPORT vector) and *Renilla* luciferase (from the phRL-TK vector) activities in the cell lysates were measured using the Glomax-Multi Detection System (Promega, Madison, WI, USA). Firefly luciferase activity was normalized to *Renilla* luciferase activity. Luciferase activity was measured three times for every experimental condition.

Luciferase reporter assays for *BHLHE41*, *CHMP1A*, and *NOG* were carried out in a similar manner. The primers for 3′-UTR amplification and inverse PCR are shown in Tables S2 and S3, respectively.

### 4.11. Statistics

All statistical analyses were performed using IBM SPSS Statistics (version 25; IBM, Armonk, NY, USA). The significance of between-group differences was assessed using a Student’s *t*-test or analysis of variance followed by Tukey’s test. A *p*-value < 0.05 was considered to indicate significance.

## Data Availability

RNA-sequencing data are available from the NCBI GEO database (GEO Accession No. GSE163651; https://www.ncbi.nlm.nih.gov/geo/).

## Supplementary Materials

Supplementary materials can be found at https://www.mdpi.com/1422-0067/22/3/1237/s1.

## Abbreviations

CVT	chorionic villous trophoblast
DEG	differentially expressed gene
EVT	extravillous trophoblast
FBS	fetal bovine serum
FDR	false discovery rate
lncRNA	long non-coding RNA
miRNA	microRNA
NC	negative control
ncRNA	non-coding RNA
PCR	polymerase chain reaction
siRNA	small interfering RNA
sncRNA	short non-coding RNA
UTR	untranslated region
