# Supplementary figures and images for: LncRNA H19-Derived miR-675-5p Accelerates the Invasion of Extravillous Trophoblast Cells by Inhibiting GATA2 and Subsequently Activating Matrix Metalloproteinases
Source: Int J Mol Sci. 2021 Jan 27;22(3):1237. doi: 10.3390/ijms22031237 (PMC7866107; doi:10.3390/ijms22031237)

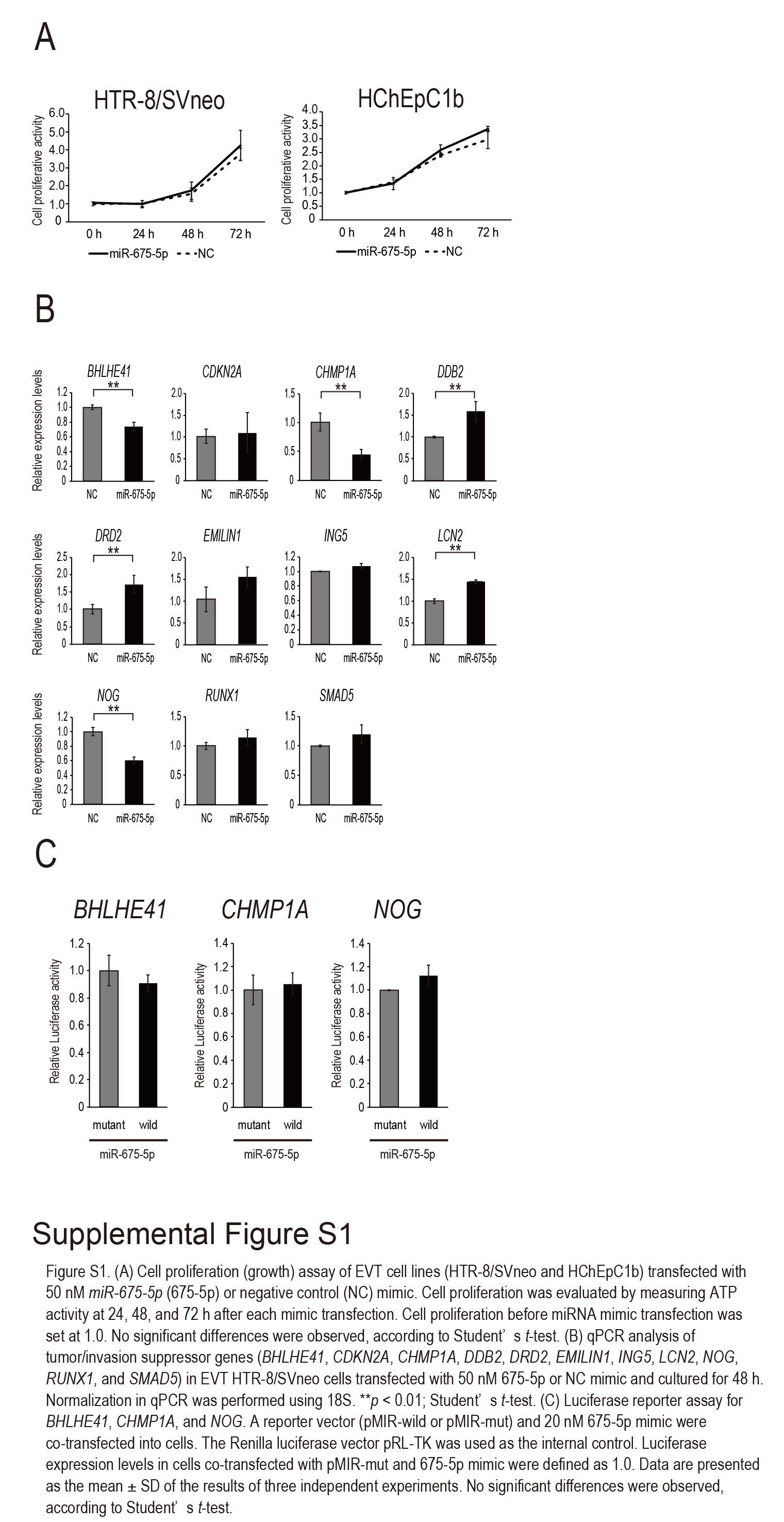

Supplement: Supplementary file 1 [file ijms-22-01237-s001.zip › 01 Suppl Fig S1 Tables S1-S11 ver210126/Fig S1 ver210126.tif]

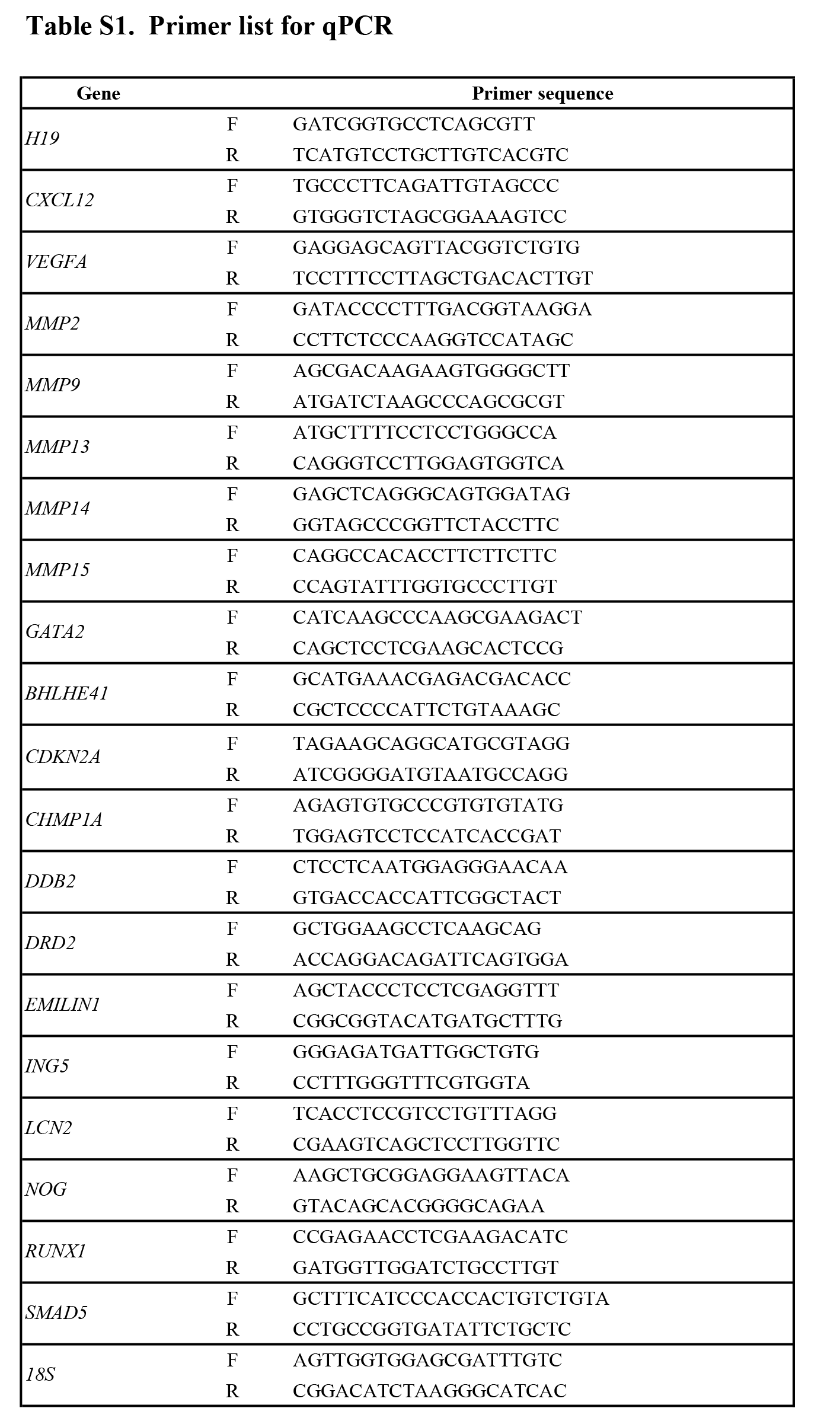

Supplement: Supplementary file 1 [file ijms-22-01237-s001.zip › 01 Suppl Fig S1 Tables S1-S11 ver210126/Table S1.tif]

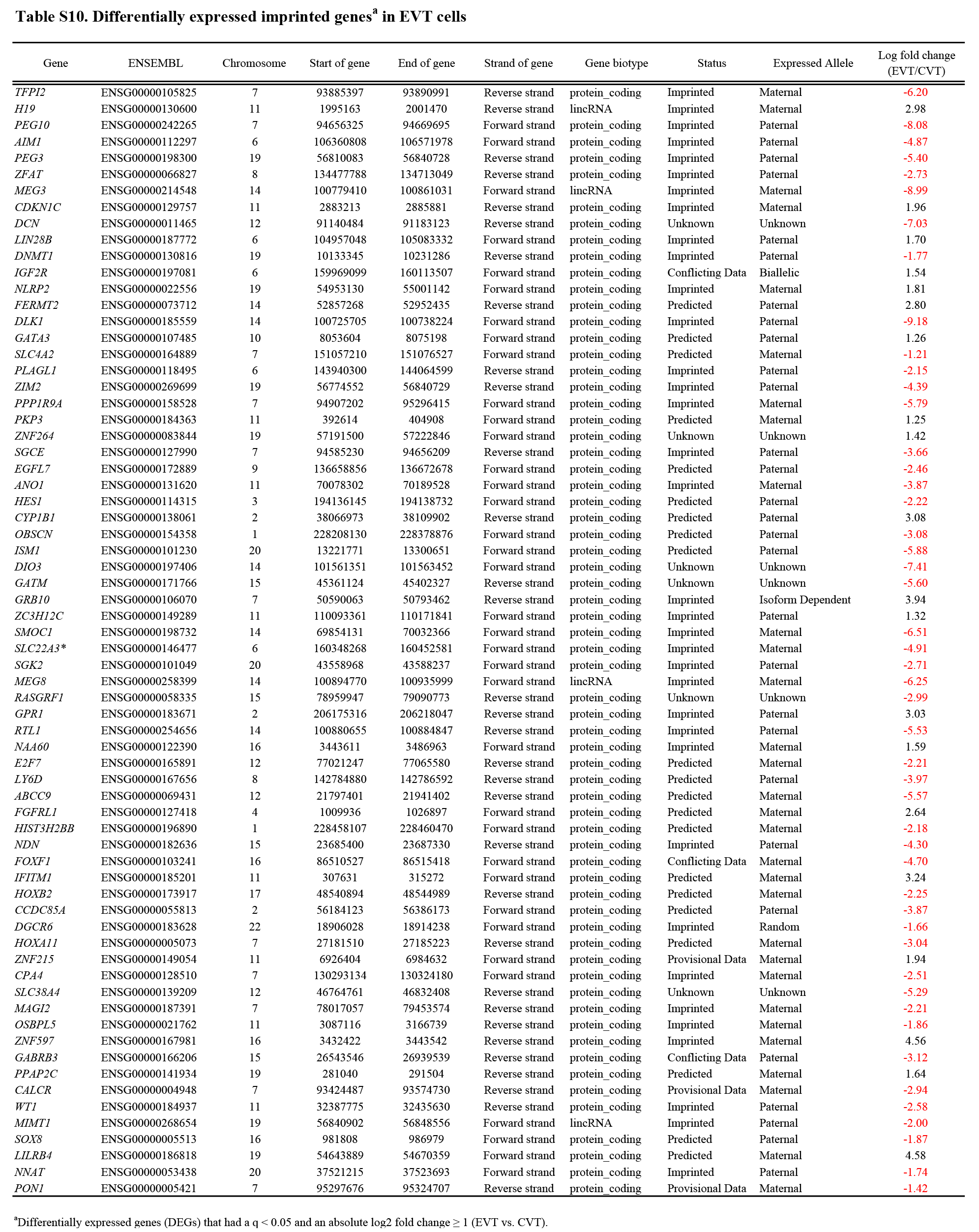

Supplement: Supplementary file 1 [file ijms-22-01237-s001.zip › 01 Suppl Fig S1 Tables S1-S11 ver210126/Table S10.tif]

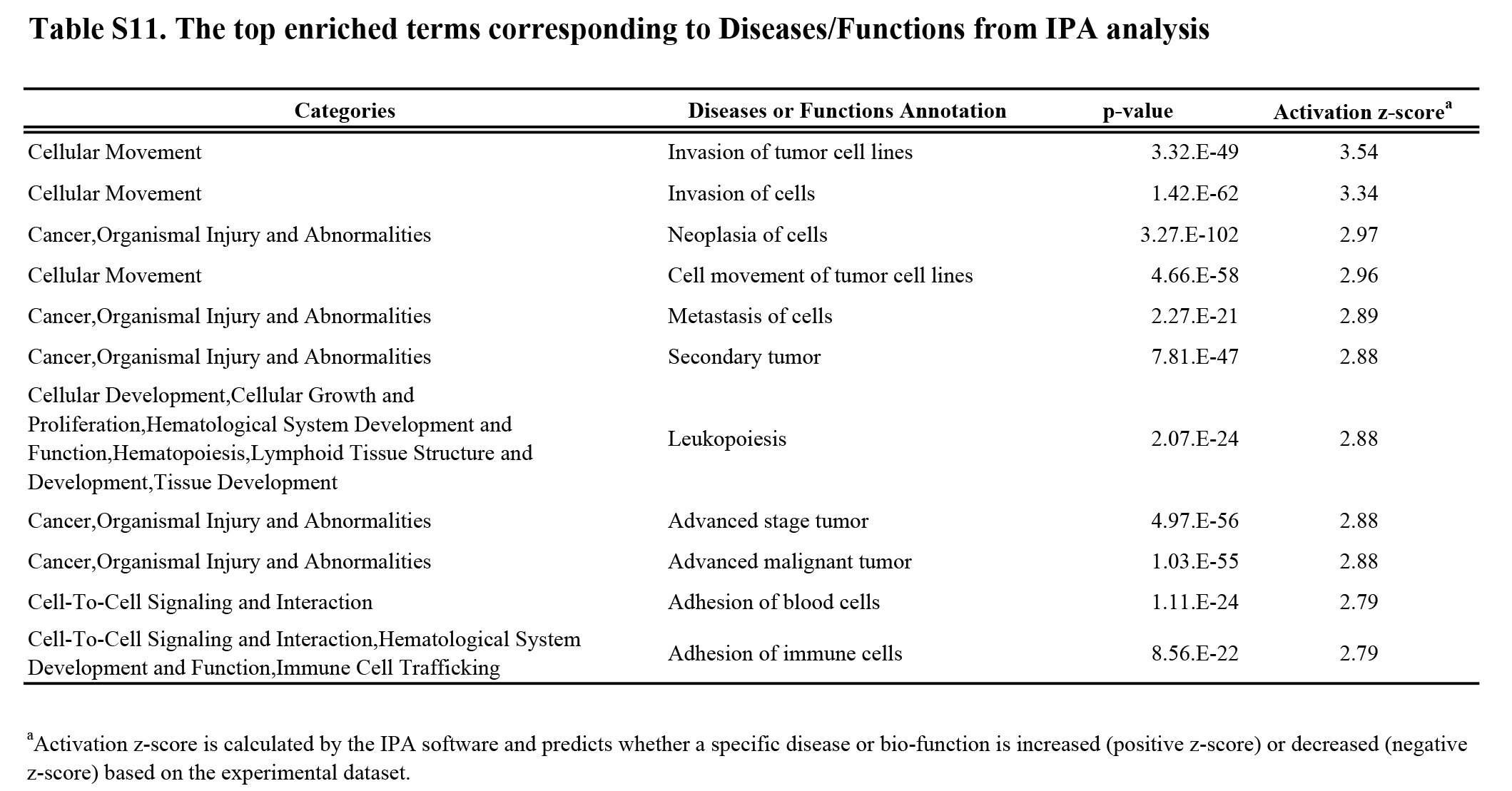

Supplement: Supplementary file 1 [file ijms-22-01237-s001.zip › 01 Suppl Fig S1 Tables S1-S11 ver210126/Table S11.tif]

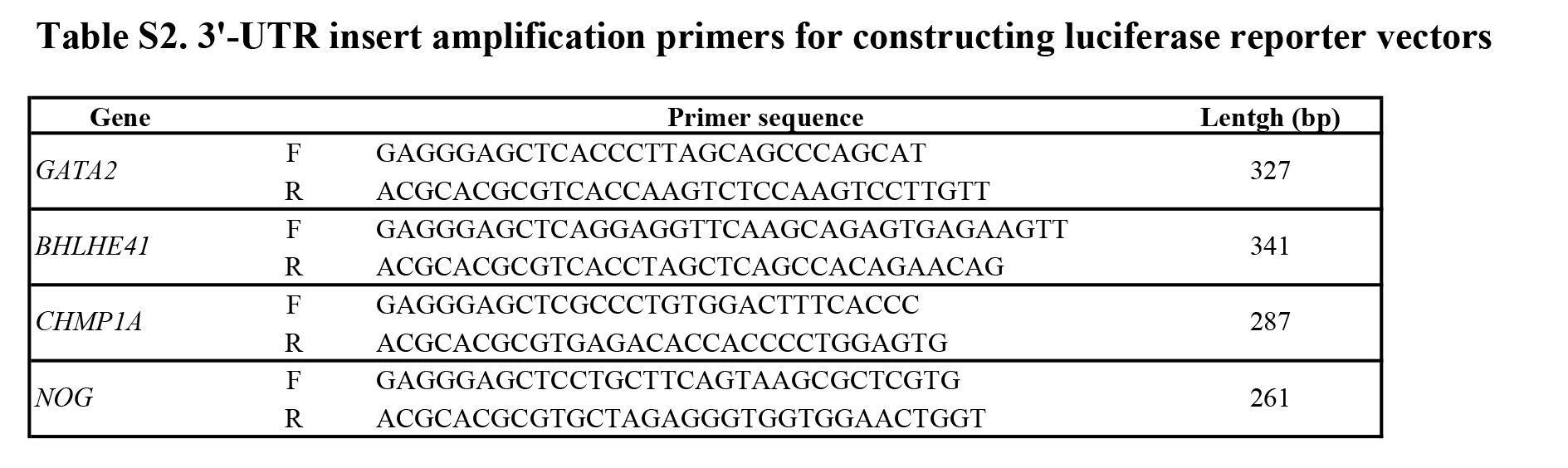

Supplement: Supplementary file 1 [file ijms-22-01237-s001.zip › 01 Suppl Fig S1 Tables S1-S11 ver210126/Table S2.tif]

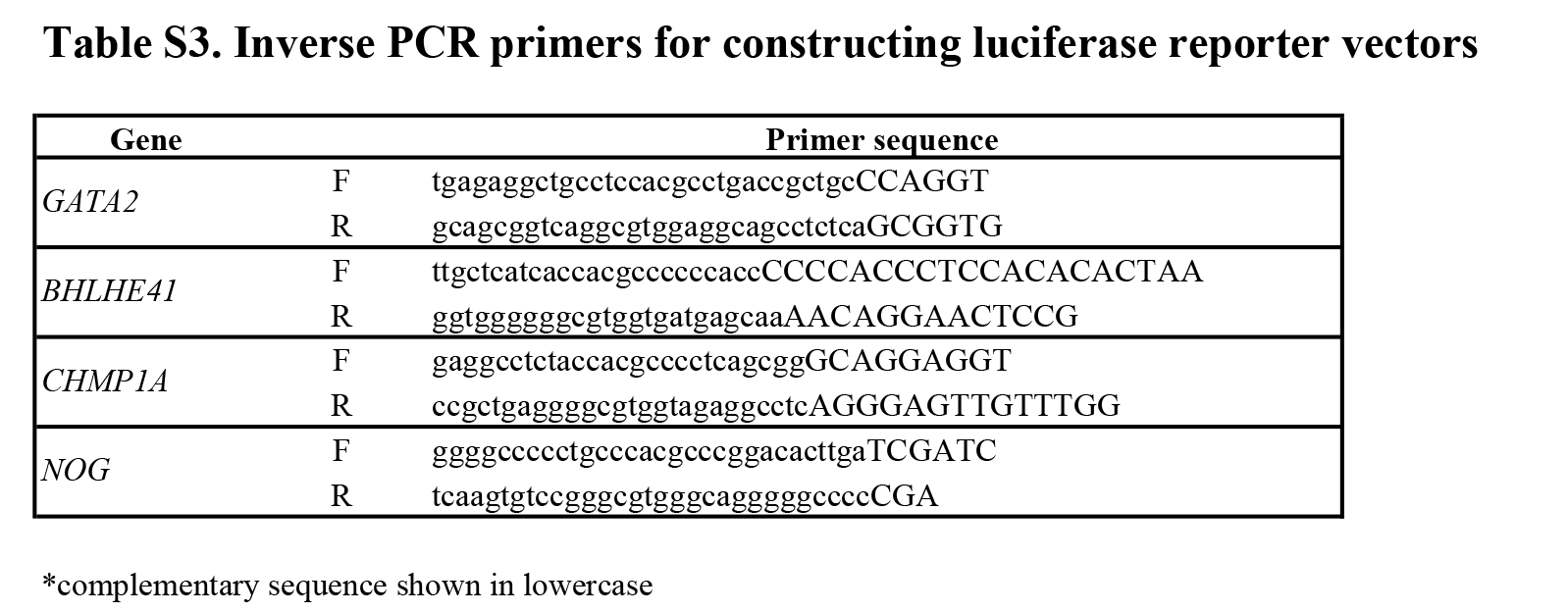

Supplement: Supplementary file 1 [file ijms-22-01237-s001.zip › 01 Suppl Fig S1 Tables S1-S11 ver210126/Table S3.tif]

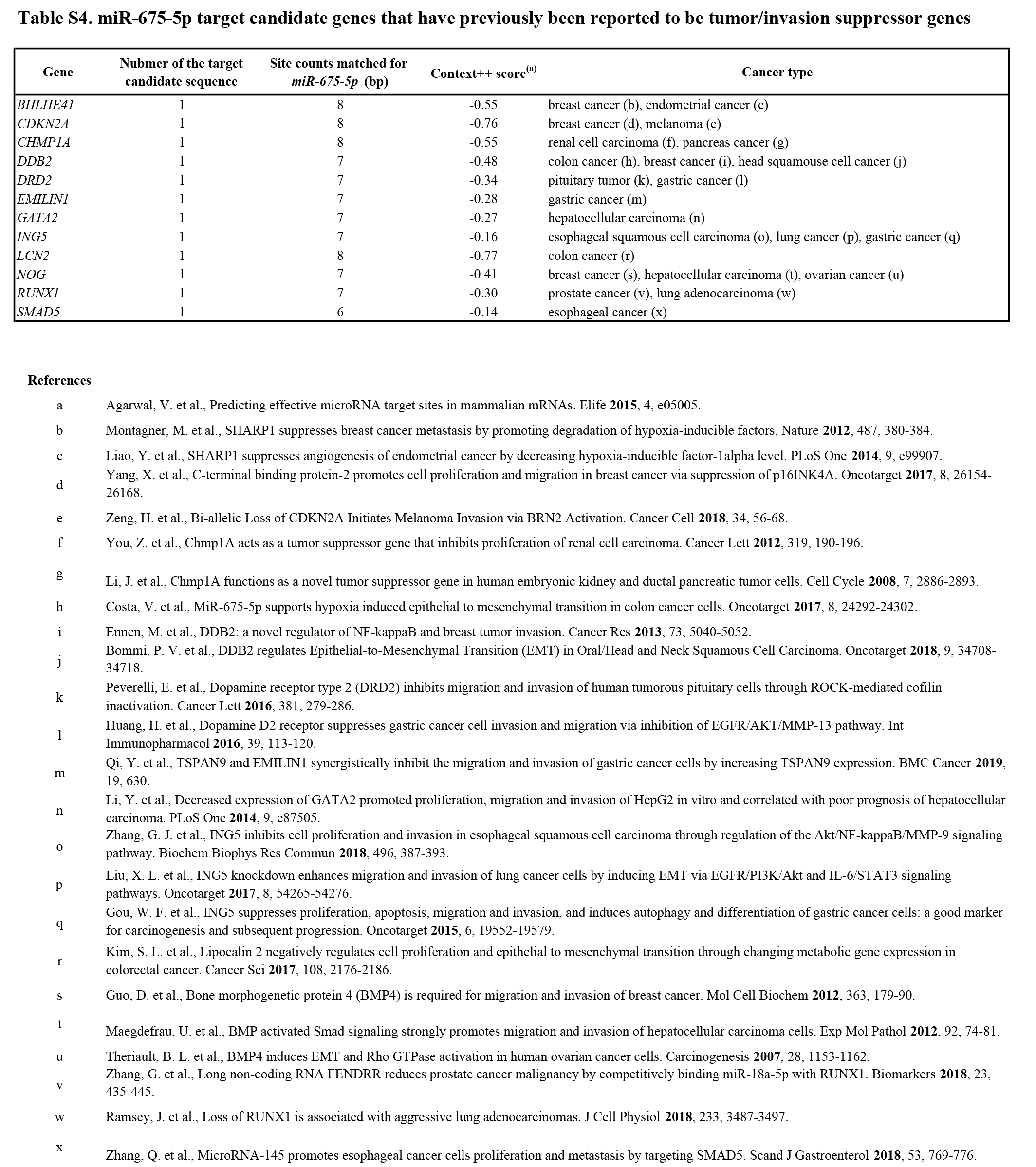

Supplement: Supplementary file 1 [file ijms-22-01237-s001.zip › 01 Suppl Fig S1 Tables S1-S11 ver210126/Table S4.tif]

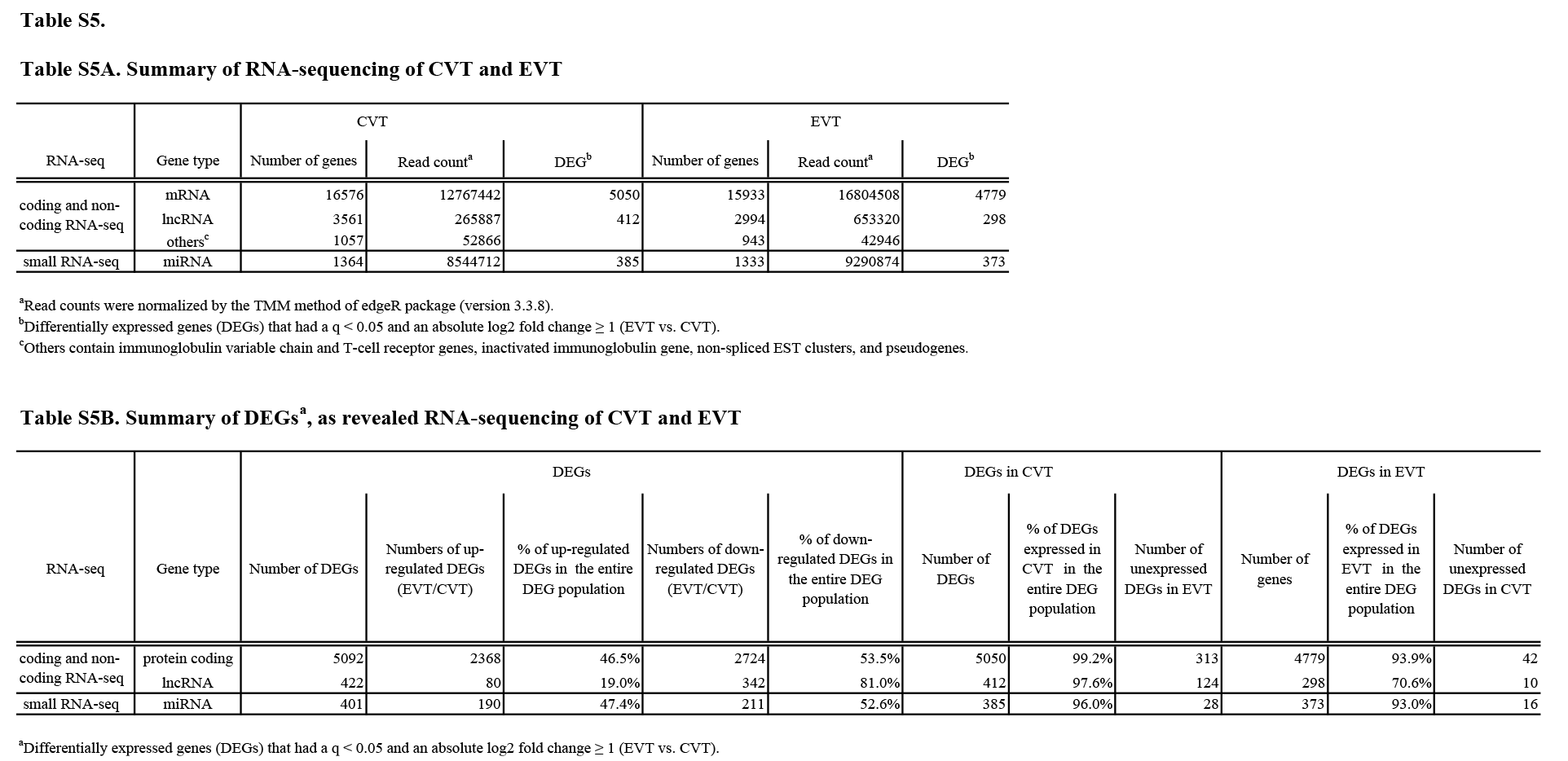

Supplement: Supplementary file 1 [file ijms-22-01237-s001.zip › 01 Suppl Fig S1 Tables S1-S11 ver210126/Table S5A and S5B verD.tif]

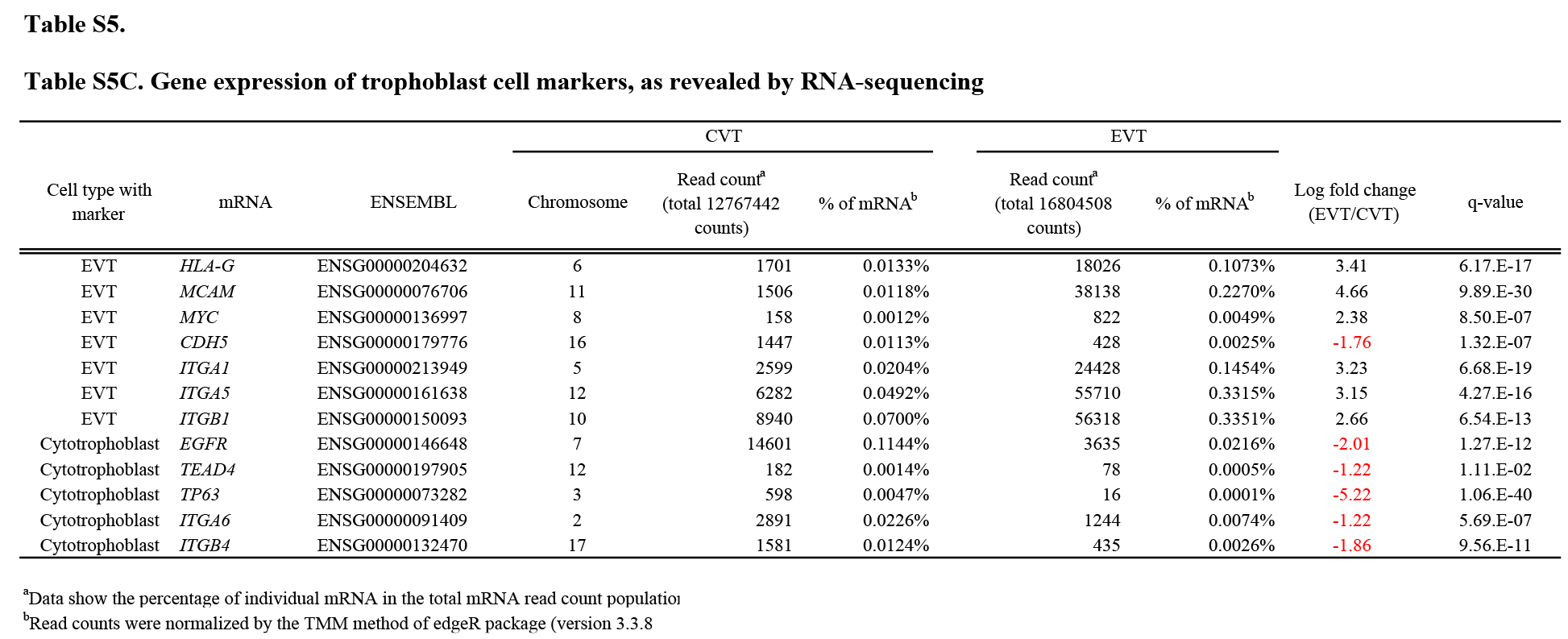

Supplement: Supplementary file 1 [file ijms-22-01237-s001.zip › 01 Suppl Fig S1 Tables S1-S11 ver210126/Table S5C.tif]

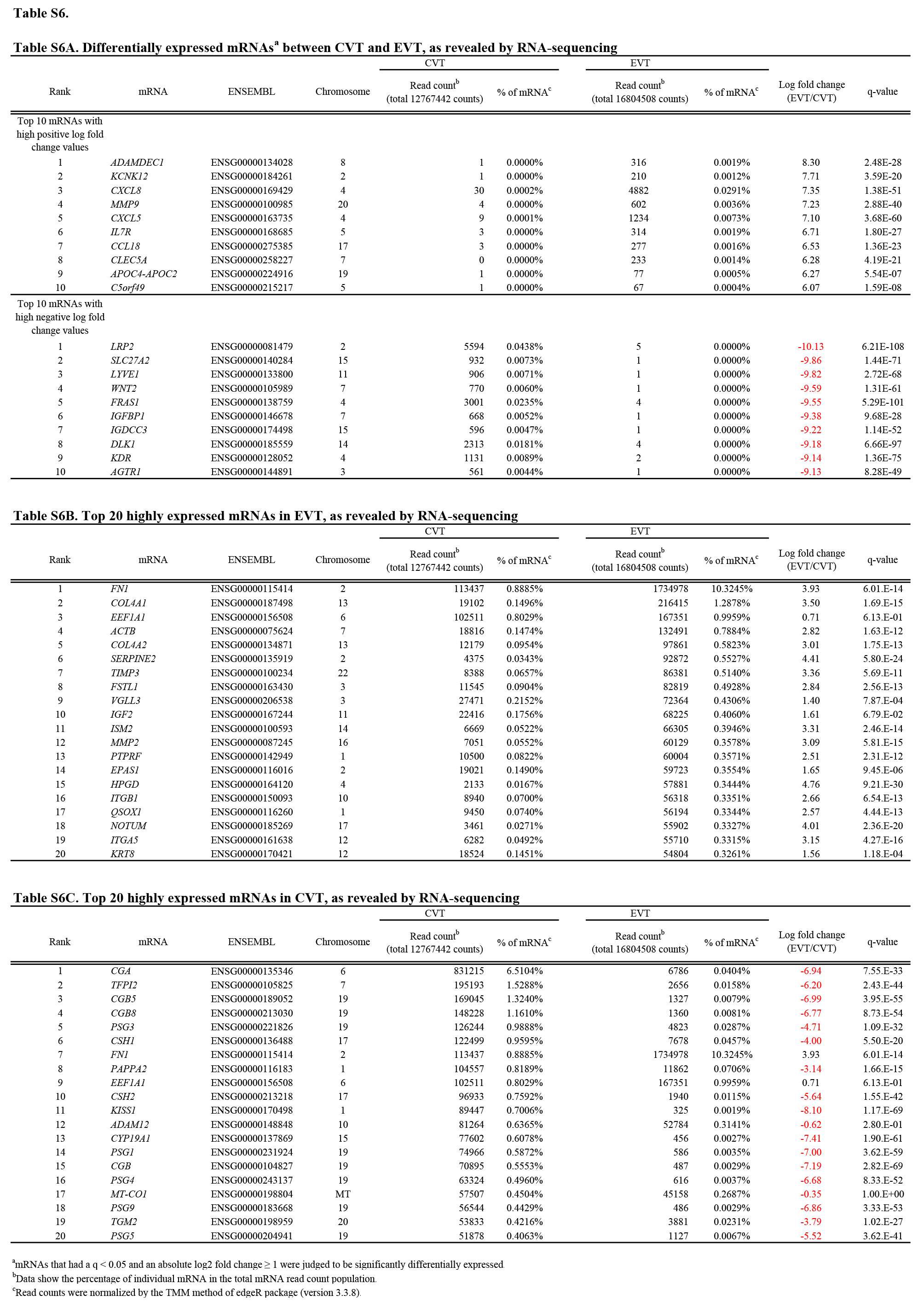

Supplement: Supplementary file 1 [file ijms-22-01237-s001.zip › 01 Suppl Fig S1 Tables S1-S11 ver210126/Table S6.tif]

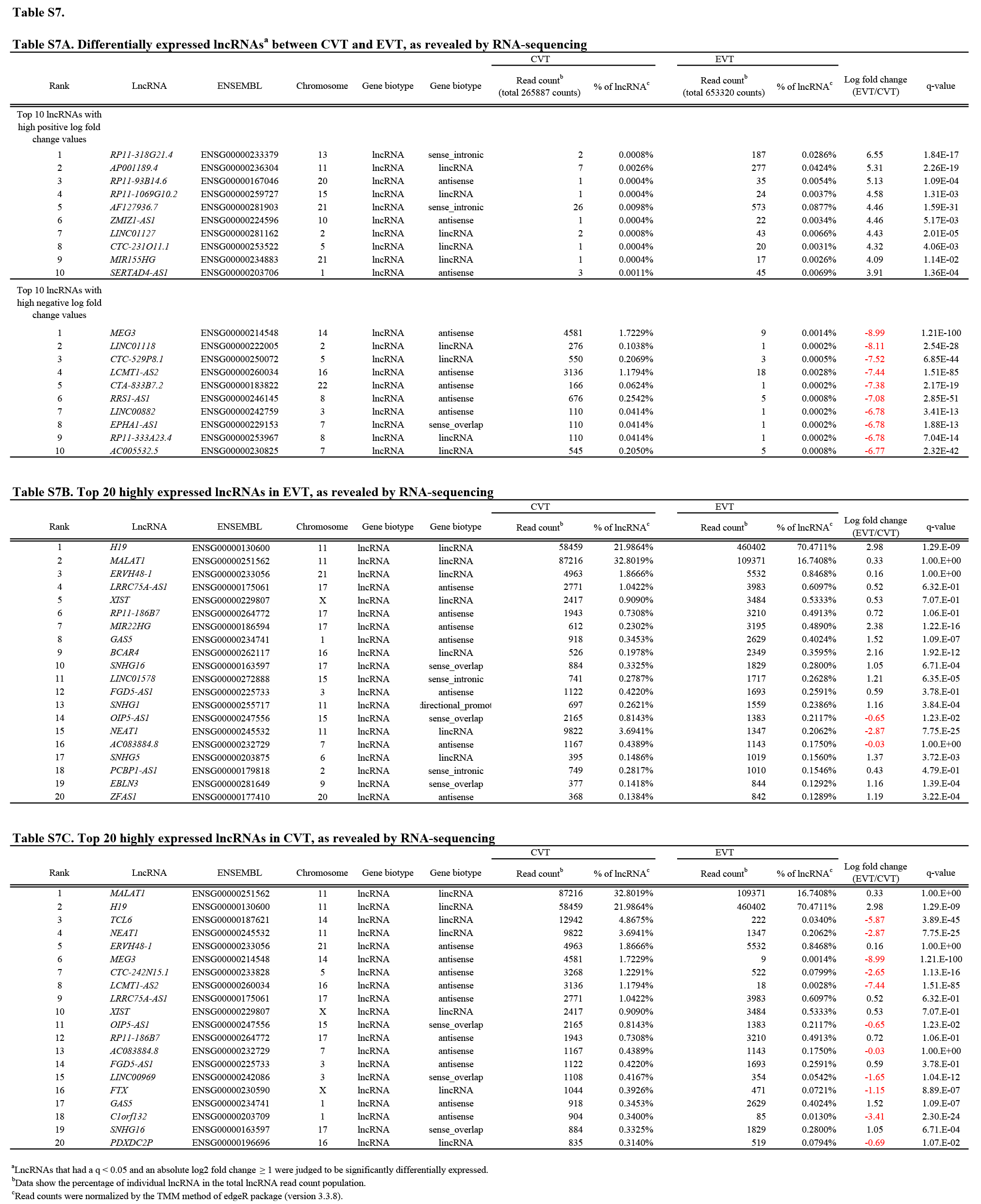

Supplement: Supplementary file 1 [file ijms-22-01237-s001.zip › 01 Suppl Fig S1 Tables S1-S11 ver210126/Table S7.tif]

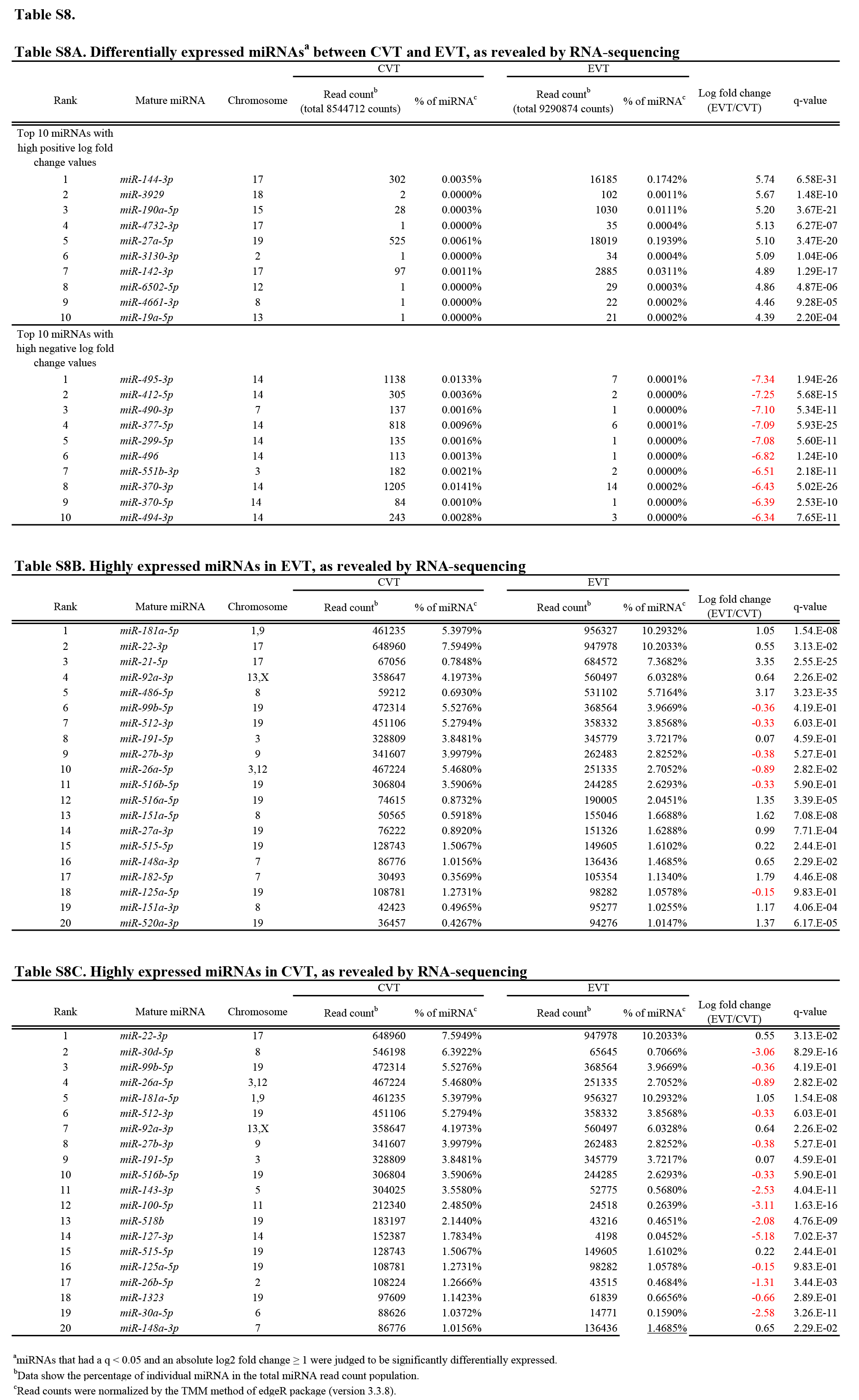

Supplement: Supplementary file 1 [file ijms-22-01237-s001.zip › 01 Suppl Fig S1 Tables S1-S11 ver210126/Table S8 verD.tif]

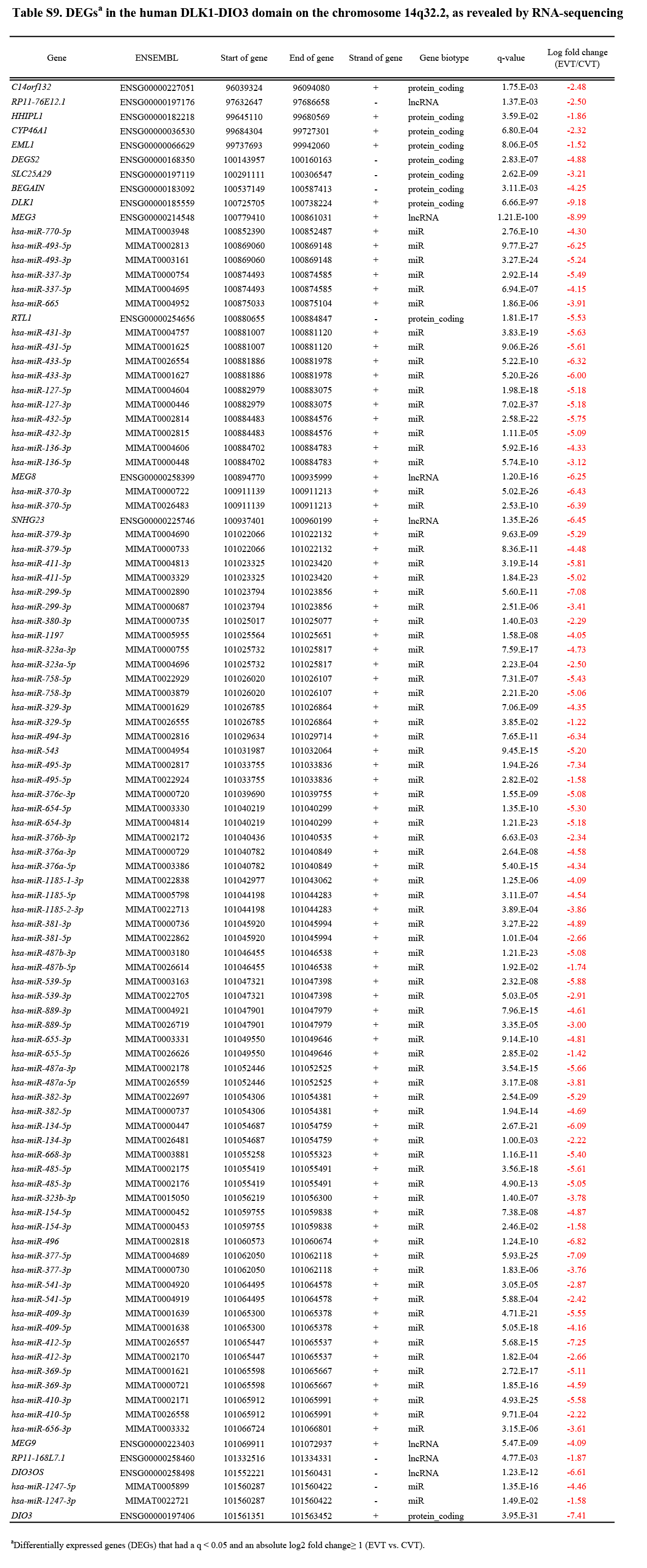

Supplement: Supplementary file 1 [file ijms-22-01237-s001.zip › 01 Suppl Fig S1 Tables S1-S11 ver210126/Table S9.tif]
